# Hyperuricemia predicts increased cardiovascular events in patients with chronic coronary syndrome after percutaneous coronary intervention: A nationwide cohort study from Japan

**DOI:** 10.3389/fcvm.2022.1062894

**Published:** 2023-01-10

**Authors:** Naoyuki Akashi, Masanari Kuwabara, Tetsuya Matoba, Takahide Kohro, Yusuke Oba, Tomoyuki Kabutoya, Yasushi Imai, Kazuomi Kario, Arihiro Kiyosue, Yoshiko Mizuno, Kotaro Nochioka, Masaharu Nakayama, Takamasa Iwai, Yoko Nakao, Yoshitaka Iwanaga, Yoshihiro Miyamoto, Masanobu Ishii, Taishi Nakamura, Kenichi Tsujita, Hisahiko Sato, Hideo Fujita, Ryozo Nagai

**Affiliations:** ^1^Division of Cardiovascular Medicine, Jichi Medical University Saitama Medical Center, Saitama, Japan; ^2^Department of Cardiology, Toranomon Hospital, Tokyo, Japan; ^3^Department of Cardiovascular Medicine, Kyushu University Graduate School of Medical Sciences, Fukuoka, Japan; ^4^Department of Clinical Informatics, Jichi Medical University School of Medicine, Tochigi, Japan; ^5^Division of Cardiovascular Medicine, Department of Medicine, Jichi Medical University School of Medicine, Tochigi, Japan; ^6^Division of Clinical Pharmacology, Department of Pharmacology, Jichi Medical University, Tochigi, Japan; ^7^Department of Cardiovascular Medicine, The University of Tokyo Hospital, Tokyo, Japan; ^8^Department of Cardiovascular Medicine, Tohoku University Graduate School of Medicine, Clinical Research, Innovation and Education Center, Tohoku University Hospital, Sendai, Japan; ^9^Department of Medical Informatics, Tohoku University Graduate School of Medicine, Sendai, Japan; ^10^Department of Cardiovascular Medicine, National Cerebral and Cardiovascular Center, Suita, Japan; ^11^Open Innovation Center, National Cerebral and Cardiovascular Center, Suita, Japan; ^12^Department of Cardiovascular Medicine, Graduate School of Medical Sciences, Kumamoto University, Kumamoto, Japan; ^13^Precision Inc., Tokyo, Japan; ^14^Jichi Medical University, Tochigi, Japan

**Keywords:** hyperuricemia, serum uric acid, chronic coronary syndrome, percutaneous coronary intervention, real-world database

## Abstract

**Background:**

The causal relationship between hyperuricemia and cardiovascular diseases is still unknown. We hypothesized that hyperuricemic patients after percutaneous coronary intervention (PCI) had a higher risk of major adverse cardiovascular events (MACE).

**Methods:**

This was a large-scale multicenter cohort study. We enrolled patients with chronic coronary syndrome (CCS) after PCI between April 2013 and March 2019 using the database from the Clinical Deep Data Accumulation System (CLIDAS), and compared the incidence of MACE, defined as a composite of cardiovascular death, myocardial infarction, and hospitalization for heart failure, between hyperuricemia and non-hyperuricemia groups.

**Results:**

In total, 9,936 patients underwent PCI during the study period. Of these, 5,138 patients with CCS after PCI were divided into two group (1,724 and 3,414 in the hyperuricemia and non-hyperuricemia groups, respectively). The hyperuricemia group had a higher prevalence of hypertension, atrial fibrillation, history of previous hospitalization for heart failure, and baseline creatinine, and a lower prevalence of diabetes than the non-hyperuricemia group, but the proportion of men and age were similar between the two groups. The incidence of MACE in the hyperuricemia group was significantly higher than that in the non-hyperuricemia group (13.1 vs. 6.4%, log-rank *P* < 0.001). Multivariable Cox regression analyses revealed that hyperuricemia was significantly associated with increased MACE [hazard ratio (HR), 1.52; 95% confidential interval (CI), 1.23–1.86] after multiple adjustments for age, sex, body mass index, estimated glomerular filtration rate, left main disease or three-vessel disease, hypertension, diabetes mellitus, dyslipidemia, history of myocardial infarction, and history of hospitalization for heart failure. Moreover, hyperuricemia was independently associated with increased hospitalization for heart failure (HR, 2.19; 95% CI, 1.69–2.83), but not cardiovascular death or myocardial infarction after multiple adjustments. Sensitive analyses by sex and diuretic use, B-type natriuretic peptide level, and left ventricular ejection fraction showed similar results.

**Conclusion:**

CLIDAS revealed that hyperuricemia was associated with increased MACE in patients with CCS after PCI. Further clinical trials are needed whether treating hyperuricemia could reduce cardiovascular events or not.

## 1. Introduction

Epidemiological studies have shown that elevated serum uric acid (SUA) levels are associated with the increase of cardiovascular events (1–4). Cardiovascular risk factors such as hypertension, diabetes mellitus (DM), and chronic kidney disease may also coexist with hyperuricemia (5–7). Hyperuricemia and cardiovascular diseases are known to be associated with both, but it is unclear whether the relationship is causal or not (8). Some studies showed the positive relationship between hyperuricemia and cardiovascular disease (9, 10), but several studies did not show the relationship (11, 12). We hypothesized that this discrepancy could be mainly from the study subjects and study design because SUA is confounded with many cardiovascular risk factors, such as diet, obesity, hypertension, DM, chronic kidney disease, and so on (13). Therefore, we conducted a large-scale, cohort study to evaluate the relationship between hyperuricemia and cardiovascular events in patients after percutaneous coronary intervention (PCI) with multiple cardiovascular risk factor adjustments.

The study was conducted to test our hypothesis that hyperuricemic patients with chronic coronary syndrome (CCS) after PCI is associated with increased risk of major adverse cardiovascular events (MACE).

## 2. Materials and methods

### 2.1. Clinical Deep Data Accumulation System (CLIDAS)

The CLIDAS, which involves seven hospitals (six university hospitals and the National Cerebral and Cardiovascular Center Hospital in Japan), obtains clinical data including patient background, laboratory data, prescriptions, echocardiographic parameters, electrocardiogram, cardiac catheterization reports, and long-term prognosis. The Standardized Structured Medical Information eXchange version 2 (SS-MIX2) standard storage is used to collect basic patient information, prescriptions, and laboratory data from electronic medical records, whereas the SS-MIX2 extended storage is used to collect the data with non-standardized formats such as physiological tests, cardiac catheterization, and cardiac catheter intervention reports (14). The development of the CLIDAS started as the Japan Ischemic heart disease Multimodal Prospective Data Acquisition for Precision Treatment project launched in 2015, aimed at creating a hospital information system (HIS)-based system that electronically collected both medical records and other clinical data in standardized data formats for clinical studies (15). In brief, HIS data, picture archiving and communication system data, and physiology data server were linked to multi-purpose clinical data repository system (MCDRS) then to the SS-MIX2 standard storage system through the firewalls (15). Each institution output data was linked from its own MCDRS server to the CLIDAS server after anonymization. Patient background information and follow-up data were collected by data managers and researchers at each site. After all data collected from each facility was aggregated into a central database, researchers retrieved the information necessary for the study and combined it based on the patient's unique anonymized ID for an integrated analysis of the data. In other fields, such as diabetes and renal disease, there are storage systems using the SS-MIX2 as well as the CLIDAS (16, 17). The collection of detailed longitudinal data on cardiovascular outcomes using SS-MIX is a unique feature of the CLIDAS.

### 2.2. Study design and population

This was a retrospective, multicenter, observational cohort study. We registered coronary artery disease (CAD) patients who had undergone PCI at seven hospitals between April 2013 and March 2019. This study period was determined as the period from the time when available data could be consistently obtained from each facility to the end of the ethics committee's accreditation. This study was approved by the Institutional Review Board of Jichi Medical University Saitama Medical Center (S21-163), and was conducted in accordance with the Declaration of Helsinki. The requirement for written informed consent was waived due to the retrospective study design. Patients with acute coronary syndrome and those with no event data were excluded. The final study population was categorized into two groups according to baseline SUA levels and/or prescribed urate-lowering drugs; the hyperuricemia group: patients with hyperuricemia at baseline, and the non-hyperuricemia group: patients without hyperuricemia at baseline.

The primary outcome was MACE, defined as a composite of cardiovascular death, myocardial infarction, or hospitalization for heart failure. The secondary outcomes were all-cause death and each component of MACE. The event-free time was calculated from the index PCI to the event date or the last follow-up date. Event confirmation was done at each facility using record information about patient visits, phone calls, and letters.

### 2.3. Definitions

In the CLIDAS database, index PCI was defined as the first PCI procedure within the study period if multiple vessels were treated in a staged manner. To increase the acquisition rate of laboratory data values, all baseline laboratory data were calculated as the average values from 60 days before the index PCI to 30 days after the procedure. In this study, hyperuricemia was defined as a SUA level ≥7.0 mg/dL for men or ≥6.0 mg/dL for women and/or taking urate-lowering drugs according to the previous studies (18–21). CCS was defined as cases of PCI other than acute coronary syndrome (22). Hypertension was defined as a systolic blood pressure ≥140 mmHg, diastolic blood pressure ≥90 mmHg, or a medical treatment for hypertension at index PCI. DM was defined as a hemoglobin A1C level ≥6.5%, casual blood glucose level ≥200 mg/dL, fasting blood glucose level ≥126 mg/dL, or medical treatment for DM at index PCI. Dyslipidemia was defined as a medical treatment for dyslipidemia at the index PCI or description of dyslipidemia on electronic medical records. We calculated the estimated glomerular filtration rate (eGFR) from the serum creatinine level, age, weight, and sex, using the following formula: eGFR = 194 × Cr^−1.094^ × age^−0.287^ (man); eGFR = 194 × Cr^−1.094^ × age^−0.287^ × 0.739 (woman) (23). We used echocardiographic findings closest to the index PCI, performed between −100 and 0 days before index PCI. Left ventricular ejection fraction (LVEF) was calculated using the modified Simpson's rule; however, the Teichholz method was used for LVEF measurement if the data of the modified Simpson's rule were missing.

The number of diseased vessels was defined as the number of coronary arteries with severe stenosis (≥75%) in the major epicardial coronary segments of the right coronary artery, left anterior descending artery, and left circumflex artery and their branch lesions that underwent PCI. The diseased left main trunk (LMT), defined as ≥75% stenosis, was counted separately. The patients were categorized according to the combination of the number of diseased vessels and LMT disease.

### 2.4. Statistical analysis

Categorical variables were presented as counts and percentages, and continuous variables were presented as mean ± standard deviation for normally distributed continuous variables or median [interquartile range (IQR)] for non-normally distributed continuous variables. Categorical variables were compared using the chi-square test or Fisher's exact test for small samples. Normally distributed continuous variables were compared between the groups using an unpaired Student's *t*-test. The Shapiro-Wilk test was performed to determine whether the continuous variables were normally distributed. Conversely, non-normally distributed continuous variables were compared using the Mann–Whitney *U* test. Event-free survival rates were calculated using the Kaplan–Meier method, and the statistical differences between the groups were calculated using the log-rank test. The patients were censored when they were lost to follow-up. Cox regression analyses were performed to clarify the determinants of MACE, all-cause death, and each component of MACE. For missing values, a complete case analysis was performed. Age, sex, body mass index, eGFR, left main (LM) disease or three-vessel disease (3VD), hypertension, DM, dyslipidemia, history of myocardial infarction, and history of hospitalization for heart failure were used as covariates in Model 1. Model 2 was performed using the covariates in Model 1 and diuretic use at baseline. Model 3 was used as a covariate in Model 2, B-type natriuretic peptide (BNP) levels, and LVEF at baseline. The missing numbers of BNP levels and LVEF were not small; thus, interpretation of Model 3 must be done carefully. Hazard ratios (HR) and 95% confidence intervals (CI) were calculated. All presented *P-*values were determined by two-sided analysis, and a *P*-value of <0.05 was considered statistically significant. *P*-values were presented without adjustment for multiple comparisons in an exploratory manner. All data were analyzed using SPSS ver. 28 for Windows (SPSS Inc., Chicago, Illinois, USA).

## 3. Results

**Figure 1:**
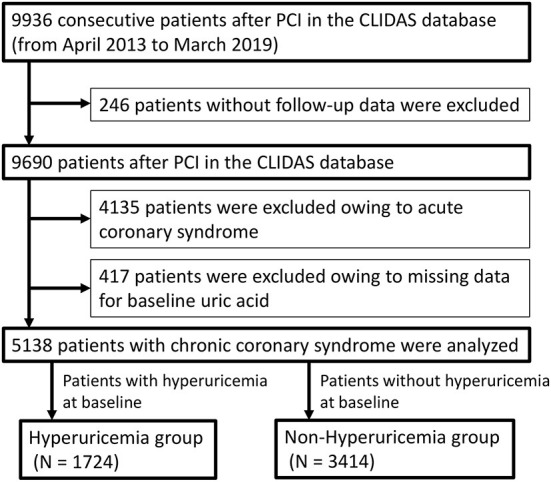
Study flow chart. PCI, percutaneous coronary intervention; CLIDAS, Clinical Deep Data Accumulation System.

A total of 9,936 consecutive patients who underwent PCI between April 2013 and March 2019 were enrolled in the CLIDAS database. Of them, 5,138 patients with CCS after PCI were analyzed. They were divided into a hyperuricemia group (*N* = 1,724) and a non-hyperuricemia group (*N* = 3,414) (Figure 1).

Table 1 shows the baseline characteristics and comparisons of the hyperuricemia and non-hyperuricemia groups. For the entire cohort, the median (IQR) age was 72 (65–78) years, and 78.4% of the participants were men. Patient age and sex were well-balanced between the two groups. The prevalence of hypertension (87.0 vs. 81.7%), atrial fibrillation (9.0 vs. 4.5%), history of coronary artery bypass grafting (8.0 vs. 6.1%), history of hospitalization for heart failure (14.7 vs. 5.6%), and baseline creatinine value [median (IQR), 1.06 (0.88–1.39) mg/dL vs. 0.85 (0.72–1.02) mg/dL] were significantly higher in the hyperuricemia group than in the non-hyperuricemia group. Conversely, the prevalence of DM (43 vs. 48%) was significantly lower in the hyperuricemia group than that in the non-hyperuricemia group. The median BNP level in the hyperuricemia group [89 (35–282) pg/mL] was higher than that in the non-hyperuricemia group [45 (21–122) pg/mL]. The LVEF was lower in the hyperuricemia group than in the non-hyperuricemia group [60.0 (44.0–67.3)% vs. 62.6 (53.4–69.0)%]. LM disease or 3VD were more frequently observed in the hyperuricemia group than in the non-hyperuricemia group (20.1 vs. 17.0%). Prescription of beta-blockers, angiotensin-converting enzyme inhibitors or angiotensin receptor blockers, diuretics, and statins was significantly higher in the hyperuricemia group than in the non-hyperuricemia group. The number of prescriptions of febuxostat and allopurinol in the hyperuricemia group was 532 (30.9%) and 326 (18.9%), respectively.

**Table 1:** Baseline characteristics between the hyperuricemia and non-hyperuricemia groups.

	**All** ** (*n* = 5,138)**	**Missing data**	**Hyperuricemia** ** (*n* = 1,724)**	**Non-hyperuricemia** ** (*n* = 3,414)**	***P*-value**
**Patient characteristics**					
Age, year, median (IQR)	72 (65–78)	0	72 (65–78)	71 (65–78)	0.42
Men, *n* (%)	4,029 (78.4)	0	1,351 (78.4)	2,678 (78.4)	0.95
Body mass index, kg/m^2^, median (IQR)	23.8 (21.8–26.3)	54 (1.1)	24.4 (22.0–27.0)	23.6 (21.6–25.9)	<0.001
Hypertension, *n* (%)	4,276 (83.5)	17 (0.3)	1,495 (87.0)	2,781 (81.7)	<0.001
Dyslipidemia, *n* (%)	4,043 (79.0)	22 (0.4)	1,371 (79.8)	2,672 (78.6)	0.33
Diabetes mellitus, *n* (%)	2,379 (46.6)	31 (0.6)	744 (43.4)	1,635 (48.2)	0.001
Atrial fibrillation, *n* (%)	308 (6.0)	17 (0.3)	154 (9.0)	154 (4.5)	0.001
Chronic kidney disease, *n* (%)	2,518 (50.0)	104 (2.0)	1,188 (70.5)	1,330 (39.7)	<0.001
Hemodialysis, *n* (%)	385 (7.5)	27 (0.5)	122 (7.1)	263 (7.7)	0.42
History of CABG, *n* (%)	345 (6.7)	20 (0.4)	138 (8.0)	207 (6.1)	0.009
History of PCI, *n* (%)	1,300 (25.4)	24 (0.5)	451 (26.3)	849 (25.0)	0.32
History of MI, *n* (%)	857 (16.8)	30 (0.6)	300 (17.5)	557 (16.4)	0.34
History of hospitalization for HF, *n* (%)	442 (8.6)	20 (0.4)	252 (14.7)	190 (5.6)	<0.001
History of stroke, *n* (%)	626 (12.2)	25 (0.5)	228 (13.3)	398 (11.7)	0.10
Systolic blood pressure on admission, mmHg, median (IQR)	127 (115–139)	86 (1.7)	126 (114–140)	127 (115–139)	0.47
Diastolic blood pressure on admission, mmHg, median (IQR)	69 (61–78)	88 (1.7)	70 (60–78)	69 (61–78)	0.29
**Laboratory data**					
Hemoglobin, g/dL, median (IQR)	12.8 (11.5–13.9)	0	12.5 (11.1–13.8)	12.9 (11.7–13.9)	<0.001
Hemoglobin A1C, %, median (IQR)	6.2 (5.8–6.8)	233 (4.5)	6.1 (5.7–6.7)	6.2 (5.8–6.9)	<0.001
Total cholesterol, mg/dL, median (IQR)	165.0 (144.0–186.0)	110 (2.1)	164.5 (143.0–185.5)	165.0 (144.5–186.3)	0.63
LDL cholesterol, mg/dL, median (IQR)	88.4 (71.8–107.2)	58 (1.1)	87.6 (71.1–106.7)	88.8 (71.9–107.5)	0.37
HDL cholesterol, mg/dL, median (IQR)	46.5 (39.0–55.7)	85 (1.7)	44.0 (37.5–53.0)	47.9 (40.0–57.0)	<0.001
Triglyceride, mg/dL, median (IQR)	120.5 (88.0–169.0)	50 (1.0)	133.3 (94.3–188.0)	114.5 (85.2–160.0)	<0.001
Creatinine, mg/dL, median (IQR)	0.91 (0.76–1.15)	107 (2.1)	1.06 (0.88–1.39)	0.85 (0.72–1.02)	<0.001
eGFR, ml/min/1.73 m^2^, median (IQR)	60.1 (45.9–72.2)	107 (2.1)	49.7 (36.6–62.6)	64.5 (53.2–75.5)	<0.001
Uric acid, mg/dL, median (IQR)	5.7 (4.8–6.6)	0	7.0 (5.8–7.7)	5.3 (4.6–6.0)	<0.001
BNP, pg/mL, median (IQR)	55 (24–170)	323 (6.3)	89 (35–282)	45 (21–122)	<0.001
**Echocardiographic finding (index** −**100 to 0 day)**					
LVEF, %, median (IQR)	61.9 (50.0–68.5)	2,451 (47.7)	60.0 (44.0–67.3)	62.6 (53.4–69.0)	<0.001
**Angiographic finding**					
LM disease or 3VD, *n* (%)	927 (18.0)	405 (7.9)	346 (20.1)	581 (17.0)	0.021
**Prescription (index** −**10 to 10 day)**					
Beta-blockers, *n* (%)	2,868 (55.8)	0	1,099 (63.7)	1,769 (51.8)	<0.001
ACE-inhibitors or ARBs, *n* (%)	2,997 (58.3)	0	1,189 (69.0)	1,808 (53.0)	<0.001
Diuretics, *n* (%)	1,174 (22.8)	0	700 (40.6)	474 (13.9)	<0.001
Statins, *n* (%)	3,955 (77.0)	0	1,395 (80.9)	2,560 (75.0)	<0.001
Febuxostat, *n* (%)	532 (10.4)	0	532 (30.9)		
Allopurinol, *n* (%)	326 (6.3)	0	326 (18.9)		
Benzbromarone, *n* (%)	65 (1.3)	0	65 (3.8)		
Probenecid, *n* (%)	3 (0.05)	0	3 (0.1)		

CABG, coronary artery bypass grafting; PCI, percutaneous coronary intervention; MI, myocardial infarction; HF, heart failure; LDL, low-density lipoprotein; HDL, high-density lipoprotein; eGFR, estimated glomerular filtration rate; BNP, B**-**type natriuretic peptide; LVEF, left ventricular ejection fraction; LM, left main; VD, vessel disease; ACE, angiotensin-converting enzyme; ARB, angiotensin II receptor blocker; IQR, interquartile range.

**Figure 2:**
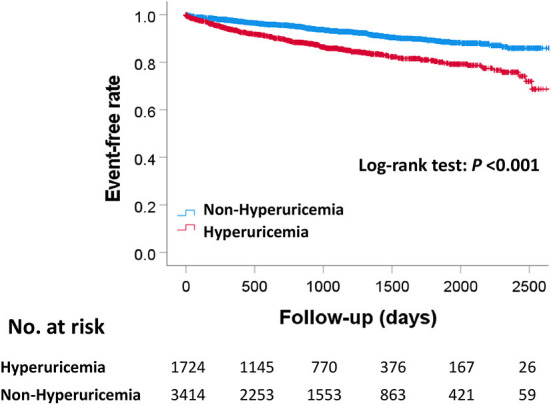
Kaplan–Meier curves for MACE between the hyperuricemia and non-hyperuricemia groups. *P*-value were calculated using the log-rank test. MACE, major adverse cardiac events.

**Figure 3:**
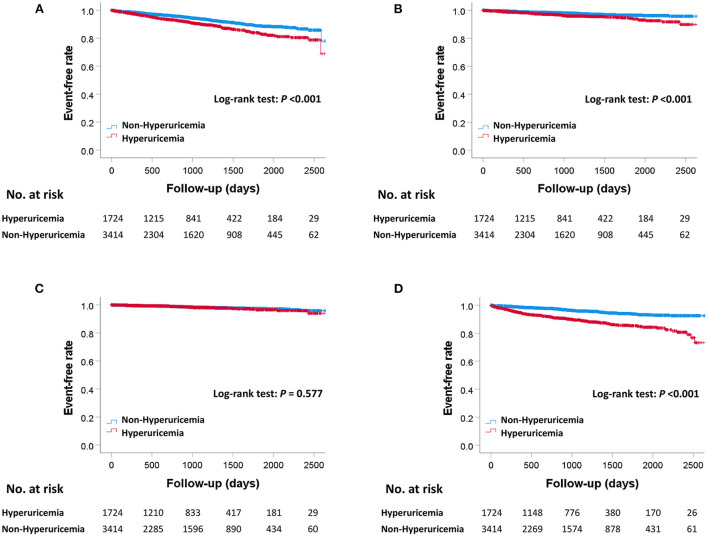
Kaplan–Meier curves for all-cause death and each component of MACE between the hyperuricemia and non-hyperuricemia groups. Kaplan–Meier curves for all-cause death **(A)**, cardiovascular death **(B)**, myocardial infarction **(C)**, and hospitalization for heart failure **(D)** between the hyperuricemia and non-hyperuricemia groups. *P*-values were calculated using the log-rank test. MACE, major adverse cardiac events.

The median follow-up duration was 910 days (307–1,479 days). During the follow-up period, there were 445 MACE, 381 all-cause deaths, 133 cardiovascular deaths, 85 myocardial infarctions, and 295 hospitalizations for heart failure (Table 2). The Kaplan–Meier curves for MACE are shown in Figure 2. The incidence of MACE was significantly higher in the hyperuricemia group than in the non-hyperuricemia group (log-rank test; *P* < 0.001). The Kaplan–Meier curves for all-cause death and each component of MACE are shown in Figure 3. The incidence of all-cause death, cardiovascular death and hospitalization for heart failure was significantly higher in the hyperuricemia group than in the non-hyperuricemia group (log-rank test; *P* < 0.001 for all of each). There was no significant difference in the prevalence of myocardial infarction between the two groups.

**Table 2:** Clinical outcomes between the hyperuricemia and non-hyperuricemia groups.

	**All** ** (*n* = 5,138)**	**Hyperuricemia** ** (*n* = 1,724)**	**Non-hyperuricemia** ** (*n* = 3,414)**
MACE, *n* (%)	445 (8.7)	226 (13.1)	219 (6.4)
All-cause death, *n* (%)	381 (7.4)	171 (9.9)	210 (6.2)
Cardiovascular death, *n* (%)	133 (2.6)	64 (3.7)	69 (2.0)
Myocardial infarction, *n* (%)	85 (1.7)	31 (1.8)	54 (1.6)
Hospitalization for heart failure, *n* (%)	295 (5.7)	173 (11.3)	122 (3.6)

MACE, major adverse cardiovascular events.

SUA levels were significantly different between men and women. Therefore, we conducted additional sensitivity analyses based on sex. The results of survival time analyses performed separately for men and women were generally consistent, except for cardiovascular death in women, which did not reach significance (log-rank test; *P* = 0.211).

The Cox regression analysis results are presented in Table 3. The hyperuricemia group was significantly associated with increased MACE in Models 1, 2, and 3 (Model 1: HR, 1.52, 95% CI, 1.23–1.86, *P* < 0.001; Model 2: HR, 1.31, 95% CI, 1.06–1.62, *P* = 0.012; Model 3: HR, 1.33, 95% CI, 1.01–1.77, *P* = 0.046). The hyperuricemia group was also significantly associated with increased hospitalization for heart failure in Models 1, 2, and 3 (Model 1: HR, 2.19, 95% CI, 1.69–2.83, *P* < 0.001; Model 2: HR, 1.76, 95% CI, 1.35–2.29, *P* < 0.001; Model 3: HR, 1.71, 95% CI, 1.21–2.41, *P* = 0.003). All-cause death was significantly associated with hyperuricemia only in model 1. Neither cardiovascular death nor myocardial infarction were significantly associated with hyperuricemia between the two groups after multiple adjustments for covariates.

**Table 3:** Cox regression analysis predicting MACE, all-cause death, and components of MACE.

**Composite endpoint**	**Hazard ratio**	**95% confidence interval**	***P*-value**
**MACE**			
Non-hyperuricemia	Reference		
Unadjusted hyperuricemia	2.10	1.74–2.52	<0.001
Adjusted hyperuricemia (model 1)	1.52	1.23–1.86	<0.001
Adjusted hyperuricemia (model 2)	1.31	1.06–1.62	0.012
Adjusted hyperuricemia (model 3)	1.33	1.01–1.77	0.046
**Component endpoints**	**Hazard ratio**	**95% confidence interval**	***P*** **-value**
**All-cause death**			
Non-hyperuricemia	Reference		
Unadjusted hyperuricemia	1.60	1.31–1.96	<0.001
Adjusted hyperuricemia (model 1)	1.26	1.01–1.57	0.044
Adjusted hyperuricemia (model 2)	1.15	0.92–1.45	0.23
Adjusted hyperuricemia (model 3)	1.13	0.82–1.56	0.47
**Cardiovascular death**			
Non-hyperuricemia	Reference		
Unadjusted hyperuricemia	1.83	1.30–2.57	<0.001
Adjusted hyperuricemia (model 1)	1.20	0.82–1.74	0.35
Adjusted hyperuricemia (model 2)	1.05	0.72–1.55	0.80
Adjusted hyperuricemia (model 3)	1.00	0.59–1.71	0.99
**Myocardial infarction**			
Non-hyperuricemia	Reference		
Unadjusted hyperuricemia	1.13	0.73–1.76	0.58
Adjusted hyperuricemia (model 1)	0.83	0.51–1.33	0.44
Adjusted hyperuricemia (model 2)	0.87	0.53–1.41	0.56
Adjusted hyperuricemia (model 3)	0.89	0.45–1.76	0.74
**Hospitalization for heart failure**			
Non-hyperuricemia	Reference		
Unadjusted hyperuricemia	2.89	2.29–3.65	<0.001
Adjusted hyperuricemia (model 1)	2.19	1.69–2.83	<0.001
Adjusted hyperuricemia (model 2)	1.76	1.35–2.29	<0.001
Adjusted hyperuricemia (model 3)	1.71	1.21–2.41	0.003

The Cox regression analysis was performed using the following covariates: Model 1 including age, sex, body mass index, eGFR, left main disease or three-vessel disease, hypertension, diabetes mellitus, dyslipidemia, history of myocardial infarction, history of hospitalization for heart failure. Model 2 including covariates in Model 1 and diuretics use at baseline. Model 3 including covariates in Model 2, BNP levels and LVEF at baseline. BNP and LVEF were entered as the categorical variables (BNP ≥100 pg/mL or not, LVEF ≥50% or not, respectively). MACE, major adverse cardiovascular events.

Since our results showed that hospitalization for heart failure events have the greatest impact on MACE, we performed additional sensitivity analyses in patients without a history of heart failure (*n* = 4676). The incidence of hospitalization for heart failure was significantly higher in the hyperuricemia group than in the non-hyperuricemia group after multiple adjustments (Model 1: HR, 2.14, 95% CI, 1.58–2.90, *P* < 0.001; Model 2: HR, 1.68, 95% CI, 1.23–2.30, *P* = 0.001; Model 3: HR, 1.61, 95% CI, 1.06–2.44, *P* = 0.026) (Supplementary Figure 1 and Supplementary Table 1).

## 4. Discussion

This large-scale, multicenter, observational cohort study revealed that the hyperuricemic patients with CCS after PCI had two times higher incidence of MACE than those without hyperuricemia during a median follow-up of 910 days. Even after multiple adjustments, hyperuricemia was independently associated with higher risk for MACE (Model 1: HR, 1.52; Model 2: HR, 1.31; Model 3: HR, 1.33). The sensitivity analyses after multiple adjustments showed that hyperuricemia was independently associated with increased hospitalization for heart failure (Model 1: HR, 2.19; Model 2: HR, 1.76; Model 3: HR, 1.71), but not cardiovascular death and myocardial infarction. These results suggest that hyperuricemia in patients with CCS after PCI could be a risk predictor for MACE, especially for heart failure.

The strengths of the study are that we adjusted many confounding factors of SUA by multiple models. SUA is associated with many cardiovascular risk factors, including age, sex, body mass index, eGFR, hypertension, DM, and dyslipidemia, and we carefully adjusted these components. Furthermore, diuretics, like thiazide and loop diuretics, increase SUA by reducing urine urate excretion, and we adjusted diuretic use in Model 2. The HR of MACE in Model 2 was less than that in Model 1 (1.31 vs. 1.52), which showed that conducting analyses by multiple models is important to remove the confounding factors associated with SUA. Even after adjustments with diuretics use, every model showed that hyperuricemia was independently associated with increased MACE. Moreover, the HR of hospitalization for heart failure in Model 2 was also lower than that in Model 1 (1.76 vs. 2.19), which suggests that SUA and diuretics are associated with each other during hospitalization for heart failure. Diuretic use is recommended for managing heart failure based on guidelines (24, 25). Our results suggests that we should take care of SUA levels in use of diuretics for heart failure as a competing risk for hospitalization for heart failure.

SUA levels were largely different between men and women, and we conducted every analysis stratified by sex. The results of survival time analyses showed that hyperuricemia was independently associated with increased cardiovascular death in men, but not in women. However, the HR in women were similar with that in men, and both HRs were more than 1. Therefore, the difference could be due to the differences in the number of patients. In fact, there were significant differences both men and women in all-cause death, which has a higher number of events than cardiovascular disease. The number of women patients was less than one-third of that of men patients. Previous studies reported that hyperuricemia in women is more risk than that in men (13, 26). We consider that the difference could be mainly due to less statistical power in women.

As several studies demonstrated, higher SUA levels were associated with the incidence of cardiovascular events in patients with CAD (9, 27–29). One multicenter, prospective observational study reported that elevated SUA level was an independent predictor of cardiovascular events and all-cause mortality in patients who had coronary artery stenosis ≥75% by coronary angiography in at least one branch of the coronary arteries (9). During the 3-years follow-up, the HR for all events, defined as cardiovascular events and all-cause mortality, was 1.25 (95% CI, 1.07–1.45) in the highest SUA quartile (SUA ≥6.8 mg/dL) after adjusting for covariates. Although these components of the composite endpoint were slightly different from those in our study, elevated SUA levels were consistently associated with increased adverse events. However, some previous studies demonstrated that increased SUA levels were not significantly associated with a higher rate of cardiovascular mortality in patients with CAD (30, 31). One prospective observational study from the Stable Coronary Artery Diseases Registry (START) registry in Italy reported that high SUA levels did not significantly influence 1-year cardiovascular events in patients with CCS with or without PCI (30). Contrary to our findings, the START registry showed no relationship between SUA levels and cardiovascular events. The above discordance may be due to the fact that the follow-up period in the START registry (1 year) was shorter than our follow-up period (median 910 days). In fact, the highest SUA tertile group (SUA ≥6.23 mg/dL) tended to have an increased incidence of MACE, including cardiovascular death and hospitalization for myocardial infarction, heart failure, angina, or revascularization, suggesting that a longer follow-up period might have revealed a significant relationship between increased SUA levels and MACE in the START registry (30).

We should discuss why MACE, especially hospitalization for heart failure, increased in the hyperuricemia group compared with the non-hyperuricemia group. Our results revealed that the presence of hyperuricemia during PCI procedures in patients with CCS after PCI was an independent predictor of hospitalization for heart failure, whereas there was no independent relationship between hyperuricemia and cardiovascular events, including cardiovascular death and myocardial infarction. Additionally, we performed sensitivity analyses to determine whether hyperuricemia affects hospitalization for heart failure in patients without a history of heart failure (*n* = 4676), and the results showed that hyperuricemia was still associated with hospitalization for heart failure after multiple adjustments (Supplementary Table 1). It has been reported that uric acid metabolism plays an important role in the development of cardiovascular disease, especially in the early stages of cardiovascular disease (32). In the CARDIA study, a cohort of young subjects showed that the elevation of SUA levels might be a biomarker for early atherosclerosis, as assessed by coronary artery calcified plaque and carotid intima-media thickness (33). A recent study using dual-energy computed tomography detected coronary monosodium uric acid crystal deposition in gout patients (34). However, some studies showed that higher SUA levels were not significantly linked to a higher rate of cardiovascular mortality in CAD patients. These findings suggest that hyperuricemia may contribute to CAD progression in the early stages of atherosclerosis. Despite these findings, our results showed no statistically significant difference in myocardial infarction between the two groups, suggesting that hyperuricemia may contribute to heart failure events more than atherosclerotic disease progression in patients with CCS after PCI. Even after adding covariates such as diuretic use at baseline, BNP level, and LVEF at baseline, hyperuricemia was consistently associated with MACE and hospitalization for heart failure. One meta-analysis reported that every 1 mg/dL increase in SUA increases the risk of developing heart failure by 19% (HR 1.19, 95% CI 1.17–1.21) (35). Moreover, some retrospective observational studies have shown that elevated SUA levels are associated with a higher risk of heart failure in patients with CAD (36, 37). The mechanism by which uric acid influences the development of heart failure is thought to be the result of oxidative stress caused by xanthine oxidase-derived elevated uric acid and reactive oxygen species (38). Other factors, such as the renin-angiotensin-aldosterone system and the use of diuretics (39), have not been fully elucidated.

The clinical implications of this study are as follows. We revealed that the presence of hyperuricemia in patients with CCS after PCI was associated with increased MACE, especially hospitalization for heart failure. Some recent clinical guidelines have shown that urate-lowering drugs may be associated with a reduction in the risk of cardiovascular events in patients with hyperuricemia and gout (40); however, it remains unclear whether urate-lowering drugs could prevent the subsequent development of cardiovascular disease among CCS patients. The results of two randomized controlled trials on the effects of the urate-lowering drugs oxypurinol (41) and allopurinol (42) on cardiovascular events in patients with symptomatic heart failure and reduced LVEF did not show improved clinical status. Although not significant, taking allopurinol tended to reduce hospitalization for heart failure in the EXACT-HF study (42). Considering these results and our results, future studies are warranted to determine whether urate-lowering therapy in patients with CCS after PCI can reduce cardiovascular events, including hospitalization for heart failure.

### 4.1. Limitations

This study had some limitations that need to be addressed. First, because this was a retrospective observation study, we could not show the causal relationship between hyperuricemia and MACE. Further clinical trials are needed whether urate lowering treatment for hyperuricemia could reduce MACE or not. Moreover, this study has a possibility of selection bias. To reduce the selection bias, this study included all patients over the study period from seven hospitals. Second, due to the missing echocardiographic data and BNP values, these were analyzed separately in the multivariable analysis, which might lead to biased results. To address this problem, we created several models using a multivariable analysis. The results of the three models showed the same direction, and we believe that the results were reliable. Additionally, our results in Table 3 showed that hyperuricemia was significantly associated with hospitalization for heart failure, and not any other components of MACE. It is crucial to take into account echocardiographic results, such as LVEF. We performed sensitivity analyses of only those evaluated for LVEF (*n* = 2,687), and the trend of the results did not differ from that of the overall population (log-rank test for MACE, *P* < 0.001; that for cardiovascular death, *P* = 0.005; that for myocardial infarction, *P* = 0.489; that for heart failure, *P* < 0.001). Third, our database does not contain information about the history of gout; therefore, we have a mix of symptomatic and asymptomatic hyperuricemia patients. Although patients taking urate-lowering drugs may have a history of gout, survival time analysis of MACE in the hyperuricemia group according to the presence or absence of urate-lowering drugs showed no significant difference (log-rank test; *P* = 0.232). Fourth, in this study, all baseline laboratory data were calculated as the average values from 60 days before the index PCI to 30 days after the procedure, and it had time gap between the sample collection and the index PCI procedure. Therefore, the time gap could cause the associations significantly impacted. However, it is also important to increase the acquisition rate of laboratory data values, and this study adopted this methodology. Fifth, the definition of LM disease generally used a significant stenosis as ≥50% for LM. In our study, we used the definition of LM disease as ≥75% for LM when making the study protocol following the previous study (43). We have to take account for the difference of the definition of LM disease between our study and most international studies. Sixth, this study could not get the information of food, alcohol and fructose intake. SUA levels are affected by alcohol and fructose consumption. It is a limitation in a nature of this retrospective study.

## 5. Conclusion

The CLIDAS study showed that hyperuricemia was associated with an increased risk of MACE, especially increased hospitalization for heart failure, in patients with CCS after PCI. Further intervention studies are needed to determine whether urate-lowering treatment could prevent MACE in patients with CCS.

## Data availability statement

The raw data supporting the conclusions of this article will be made available by the authors, without undue reservation.

## Ethics statement

The studies involving human participants were reviewed and approved by the Institutional Review Board of Jichi Medical University Saitama Medical Center (S21-163). Written informed consent for participation was not required for this study in accordance with the national legislation and the institutional requirements.

## Author contributions

NA and HF contributed to conception and design of the study. NA, TM, TKo, TKa, MI, TN, and HS collected data and organized the database. NA performed the statistical analysis. NA, MK, and HF contributed to interpretation of the data and writing. All authors contributed to the critical revision and final approval of the manuscript.

## Supplementary material

Supplementary Figure 1 Kaplan–Meier curves for hospitalization for heart failure in patients without a history of heart failure.

Supplementary Table 1 Cox regression analysis predicting hospitalization for heart failure in patients without a history of heart failure.
