# Supplementary figures and images for: Hyperuricemia predicts increased cardiovascular events in patients with chronic coronary syndrome after percutaneous coronary intervention: A nationwide cohort study from Japan
Source: Front Cardiovasc Med. 2023 Jan 10;9:1062894. doi: 10.3389/fcvm.2022.1062894 (PMC9871893; doi:10.3389/fcvm.2022.1062894)

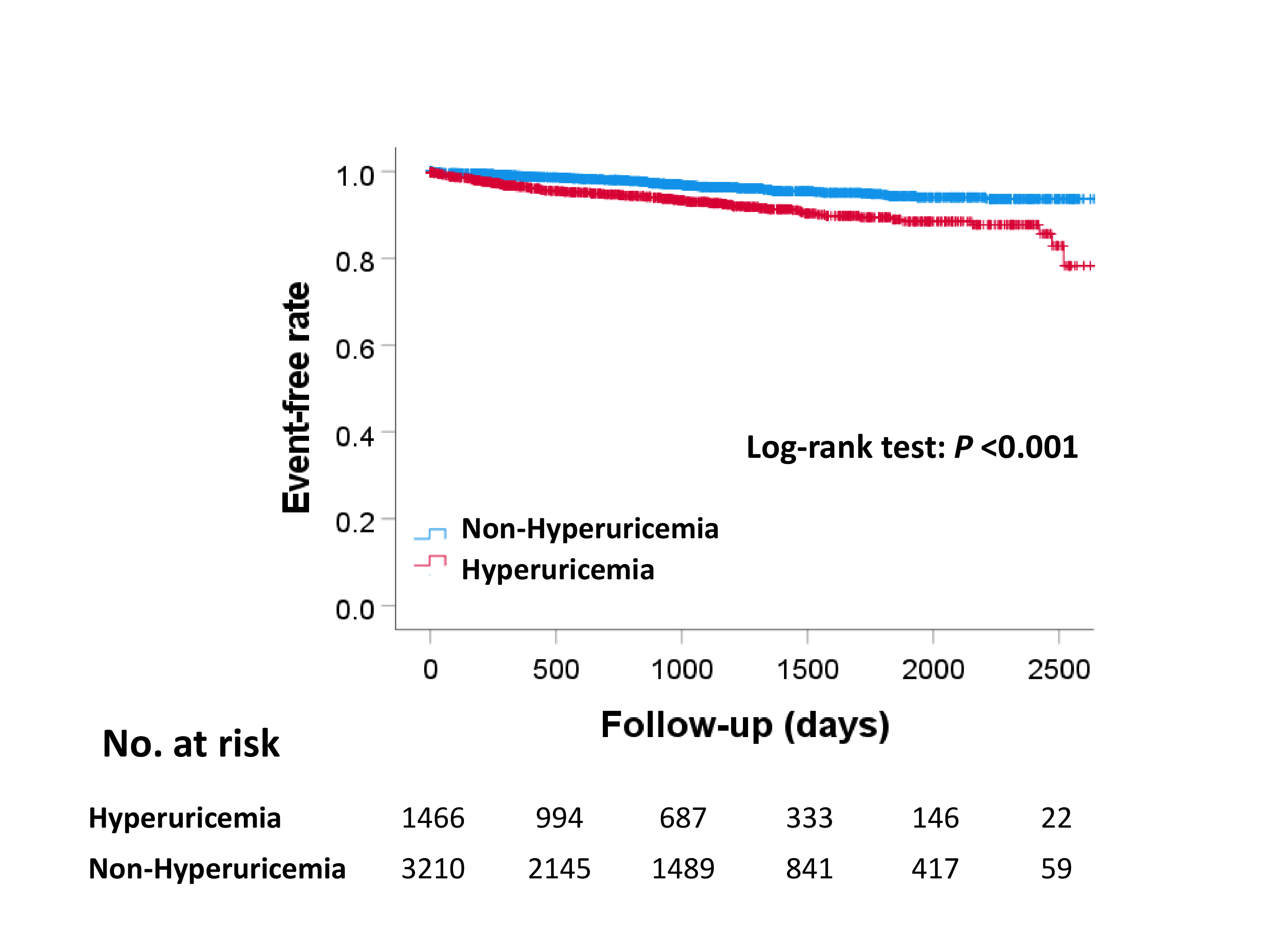

Supplement: Supplementary Figure 1 — Kaplan–Meier curves for hospitalization for heart failure in patients without a history of heart failure. [file Image_1.TIFF]
