# Supplementary material for: Hyperuricemia predicts increased cardiovascular events in patients with chronic coronary syndrome after percutaneous coronary intervention: A nationwide cohort study from Japan
Source: Front Cardiovasc Med. 2023 Jan 10;9:1062894. doi: 10.3389/fcvm.2022.1062894 (PMC9871893; doi:10.3389/fcvm.2022.1062894)
Supplement: Supplementary Table 1 — Cox regression analysis predicting hospitalization for heart failure in patients without a history of heart failure. [file Table_1.docx]

**Supplementary TABLE 1 Cox regression analysis predicting hospitalization for heart failure in patients without a history of heart failure**

|  | Hazard ratio | 95% confidence interval | *P* value |
| --- | --- | --- | --- |
| Non-Hyperuricemia | Reference |  |  |
| Unadjusted Hyperuricemia | 2.32 | 1.75-3.07 | <0.001 |
| Adjusted Hyperuricemia (Model 1) | 2.14 | 1.58-2.90 | <0.001 |
| Adjusted Hyperuricemia (Model 2) | 1.68 | 1.23-2.30 | 0.001 |
| Adjusted Hyperuricemia (Model 3) | 1.61 | 1.06-2.44 | 0.026 |

The Cox regression analysis was performed using the following covariates: Model 1 including age, sex, body mass index, eGFR, left main disease or three-vessel disease, hypertension, diabetes mellitus, dyslipidemia, history of myocardial infarction, history of hospitalization for heart failure. Model 2 including covariates in Model 1 and diuretics use at baseline. Model 3 including covariates in Model 2, BNP levels and LVEF at baseline. BNP and LVEF were entered as the categorical variables (BNP ≥100 pg/mL or not, LVEF ≥50% or not, respectively). MACE, major adverse cardiovascular events.
